# Myocardial salvage by succinate dehydrogenase inhibition in ischemia–reperfusion injury depends on diabetes stage in rats

**DOI:** 10.1007/s11010-021-04108-2

**Published:** 2021-03-05

**Authors:** Pernille Tilma Tonnesen, Marie Vognstoft Hjortbak, Thomas Ravn Lassen, Jacob Marthinsen Seefeldt, Hans Erik Bøtker, Nichlas Riise Jespersen

**Affiliations:** Department of Cardiology, Aarhus University Hospital, Palle Juul-Jensens Boulevard 99, 8200 Aarhus N, Denmark

**Keywords:** Cardiovascular metabolism, Ischemia–reperfusion injury, Diabetes mellitus, Mitochondrial function, Cardioprotection

## Abstract

Inhibition of succinate dehydrogenase (SDH) by Dimethyl Malonate (DiMal) reduces cardiac ischemia–reperfusion (IR) injury. We investigated the cardioprotective effect of DiMal in a rat model during advancing type 2 diabetes. Zucker Diabetic Fatty rats and lean controls were investigated corresponding to prediabetes, onset and mature diabetes. Hearts were mounted in an isolated perfused model, and subjected to IR for investigation of infarct size (IS) and mitochondrial respiratory control ratio (RCR). DiMal was administered for 10 min before ischemia. Compared with age-matched non-diabetic rats, prediabetic rats had larger IS (49 ± 4% vs. 36 ± 2%, *p* = 0.007), rats with onset diabetes smaller IS (51 ± 3% vs. 62 ± 3%, *p* = 0.05) and rats with mature diabetes had larger IS (79 ± 3% vs. 69 ± 2%, *p* = 0.06). At the prediabetic stage DiMal did not alter IS. At onset of diabetes DiMal 0.6 mM increased IS in diabetic but not in non-diabetic control rats (72 ± 4% vs. 51 ± 3%, *p* = 0.003). At mature diabetes DiMal 0.1 and 0.6 mM reduced IS (68 ± 3% vs. 79 ± 3% and 64 ± 5% vs. 79 ± 3%, *p*  = 0.1 and *p* = 0.01), respectively. DiMal 0.1 mM alone reduced IS in age-matched non-diabetic animals (55 ± 3% vs. 69 ± 2% *p* = 0.01). RCR was reduced at mature diabetes but not modulated by DiMal. Modulation of SDH activity results in variable infarct size reduction depending on presence and the stage of diabetes. Modulation of SDH activity may be an unpredictable cardioprotective approach.

**Supplementary Information:**

The online version contains supplementary material available at 10.1007/s11010-021-04108-2.

## Introduction

Patients with diabetes have a high prevalence of coronary artery disease [1] and impaired clinical outcome following acute myocardial infarction despite reperfusion therapy compared to patients without diabetes [2–8]. The consequences of the coronary obstruction are predominantly determined by cardiac ischemia time but also by the reperfusion injury, which influences the extent of myocardial damage following modern reperfusion therapy of acute myocardial infarction [9]. The extent of the ischemia–reperfusion (IR) damage may depend on inherent metabolic disarrays in the heart [10–12] as well as circulating glucose levels [13, 14].

Mitochondria are metabolic key regulators and hence determinants of cell fate following IR injury [15]. Mitochondrial succinate dehydrogenase (SDH) seems to be a crucial component for controlling IR injury in the heart [16]. With increasing succinate concentration during ischemia, the augmented SDH activity rapidly oxidizes succinate, reverses electron transport and enhances reactive oxygen species (ROS) production during early reperfusion [16–18]. Blocking SDH by dimethyl malonate (DiMal) during ischemia reduces succinate accumulation and IR injury [16].

Established diabetes mellitus seems to be associated with mitochondrial dysfunction [19–21] causing increased ROS production [21–25]. Activity of the malate-aspartate shuttle [26] and the complexes of the electron transport chain are compromised in diabetic hearts [20]. The role of SDH for mediating IR injury and mitochondrial dysfunction with advancing diabetes mellitus is unknown.

We have observed that the susceptibility to IR is determined by the duration of type 2 diabetes mellitus (T2DM) as sensitivity is reduced at onset diabetes [13, 26, 27], suggesting a dependency of diabetes stage on sensitivity to IR injury. The underlying mechanisms remain unknown. We have also observed that DiMal at a concentration of 0.6 mM yields cardioprotection in rats with mature diabetes [28].

In the present study we extended our investigations to explore the potential cardioprotection by DiMal to cover three different stages of diabetes: prediabetes, onset diabetes and mature diabetes [26, 27] and study the interactions between diabetes stage, mitochondrial function and effect of DiMal. We hypothesized that rats with prediabetes, onset and mature diabetes had different susceptibilities to IR due to inherent differences in mitochondrial function at different stages of diabetes and that the cardioprotective effect of DiMal might vary due to interference with the inherent variability of mitochondrial function.

Consequently, the aims of the present study were to first investigate the cardioprotective efficacy and corresponding mitochondrial respiratory capacity at three different stages of diabetes: prediabetes, onset diabetes and mature diabetes. Secondly, to compare the effect of preischemic DiMal administration on the cardioprotective ability and mitochondrial respiratory capacity in hearts from diabetic rats at the different diabetic stages and age-matched, non-diabetic rats.

## Materials and methods

### Ethics statement

Animals were handled according to national and institutional guidelines for animal research, and all surgery was performed under anesthesia. The Danish Animal Experiments Inspectorate approved the experimental study (Authorization No. 2012-15-2934-00623 and 2018-15-0201-01446).

### Animals

In the present study, we extended our previous study in 24 weeks (mature diabetes) male Zucker diabetic fatty (ZDF) rats (homozygote (fa/fa)) and age-matched non-diabetic controls (heterozygote (fa/+)) (Charles River Laboratories, 100–400 g) [28] to also include corresponding 6 weeks (prediabetes) and 12 weeks (onset diabetes) rats. For animal ethical reasons we included 24 weeks old rats from our previous study [28] in the present study. The ZDF rats from Charles River Laboratories of 6-, 12- and 24-weeks of age have previously been thoroughly characterized through experiments in our laboratory [26, 27]. Animals were kept at a constant temperature of 23 °C with a 12 h light–dark cycle and allowed unlimited access to food and water. As recommended by the supplier the rats were fed with Purina 5008 diet. No anti-diabetic treatment was given. Prior to both experimental protocols rats were fasted 14 ± 2 h (± SD) to allow correct measurement of blood glucose.

### Study design

**Fig. 1:**
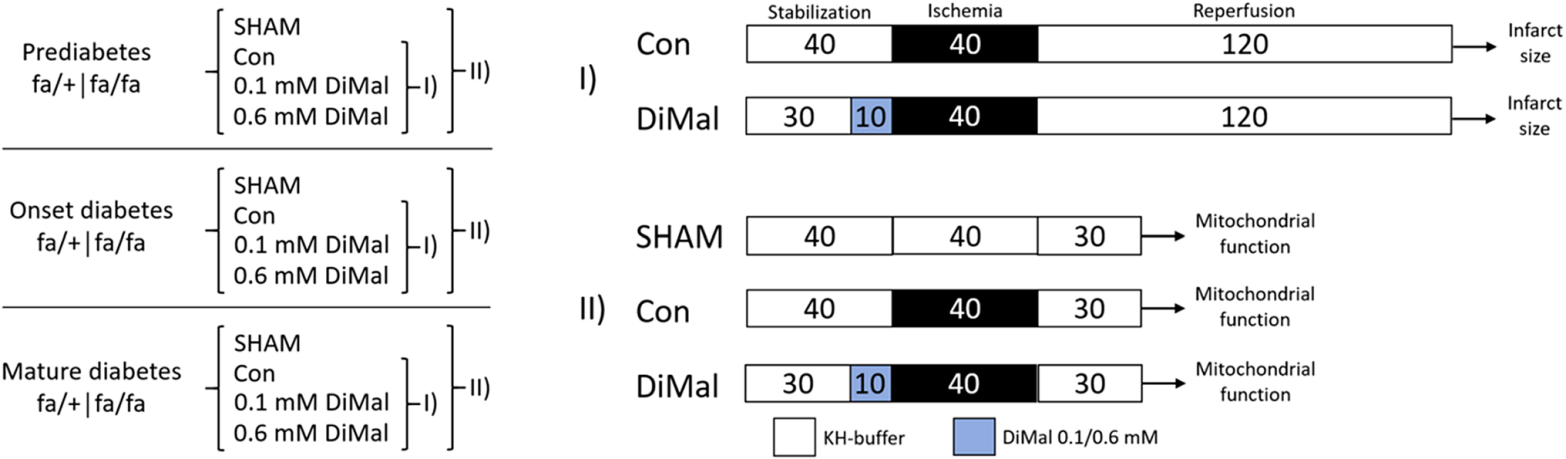
Study design and experimental protocols. Overview of groups, protocols and interventions. **I** Infarct size studies. **II** Mitochondrial function studies. Con: control. KH-buffer: Krebs Henseleit buffer

The study was divided in two experimental series: one to evaluate infarct size (series I) and one to examine mitochondrial function (series II) (Fig. 1).

### Analysis of blood glucose

Pre-anesthetic tail vein blood samples were obtained for measurements of fasting blood glucose (OneTouch® Ultra Blood Glucose, Lifescan Inc., CA, USA).

### Isolated perfused rat heart model

Rats were anesthetized by a subcutaneous injection with a mixture of Dormicum® (midazolam, 0.5 mg (kg bodyweight^−1^); Matrix Pharmaceuticals, Herlev, Denmark) and Hypnorm® (fentanyl citrate, 0.16 mg (kg bodyweight^−1^) and fluanisone, 0.5 mg (kg bodyweight^−1^); VetaPharma Limited, Leeds, United Kingdom). A tracheotomy was performed, and the rats were connected to a rodent ventilator (Ugo Basile 7025 rodentventilator, Comerio, Italy) and ventilated at 60 breaths/minute with a tidal volume of 3 mL. Subsequently, a laparotomy and thoracotomy were performed and a bolus of 1000 IU/kg heparin (Leo Pharma, Ballerup, Denmark) was administrated through the femoral vein.

The hearts were cannulated in situ and retrogradely perfused at a constant pressure of 80 mmHg with an oxygenated (95% O2 and 5% CO2) Krebs–Henseleit (KH) buffer of the following composition (mM): NaCl, 118.5; KCl, 4.7; NaHCO3, 25.0; glucose monohydrate, 11.0; MgSO4.7H2O, 1.2; CaCl2, 2.4; and KH2PO4, 1.2 (Sigma-Aldrich, St. Louis, Missouri, USA). The hearts were excised under continuous perfusion and mounted in an isolated perfused heart system (IH-SR type844/1; HSE, March-Hugstetten, Germany) where the hearts were perfused with and immersed in KH-buffer kept at a constant temperature of 37 °C throughout the study protocols [29].

After 30 or 40 min of stabilization, global no-flow ischemia was induced for 40 min (Fig. 1), which is previously reported standard procedure in our laboratory [26, 28]. Subsequently the hearts were exposed to 30 (experimental series II—mitochondrial function) or 120 (experimental series I—infarct size) minutes of reperfusion. DiMal (Dimethyl malonate 98%, Sigma-Aldrich, St. Louis, Missouri, USA) 0.1 or 0.6 mM was administered for 10 min immediately before ischemia, enhancing the probability that DiMal was also present in the coronary circulation at the beginning of reperfusion. We based our concentrations of DiMal on previous studies in our laboratory [28]. DiMal was prepared as a separate oxygenated KH-buffer solution shortly before switch of perfusion. Source of perfusion was switched back to oxygenated KH-buffer upon ischemia without inducing reperfusion of the heart.

### Hemodynamic assessment

Left ventricle isovolumetric pressure was monitored using a latex balloon (Size 7, HSE, March-Hugstetten, Germany) connected to a pressure transducer. Balloon volume was adjusted to obtain a left ventricular end-diastolic pressure of 4–8 mmHg at stabilization and not subsequently altered. Left ventricular development pressure (LVDP), heart rate, rate pressure product (RPP) and coronary flow were monitored continuously using an inline flow probe (Type 2.5SB, Transonic System Inc., Ithaca, NY, USA) (Online Resource 1).

### Infarct size

At the end of the infarct protocol hearts were frozen at − 80 °C, sliced (1.5 mm thick) and stained with 1% 2,3,5-triphenyltetrazolium chloride. Stained hearts were stored in 4% formaldehyde (Lille solution, VWR Bie & Berntsen, Herlev, Denmark) for 24–48 h to allow optimized contrast. Each slice was weighed and scanned on a high-resolution flatbed scanner (Epson Perfection V600 Photo Scanner, Epson America Inc.). Area-at-risk and infarct size were manually assessed by a blinded observer using image analysis software (ImageJ 1.46r, Wayne Rasband, National Institute of Health, USA) and adjusted to the wet weight of the individual slices. Infarct size/area-at-risk (IS/AAR) ratio was calculated for each heart and hereafter expressed as infarct size.

### Mitochondrial function assessment

At the end of the perfusion protocol in experimental series II the left ventricle (approximately 200 mg) was rapidly dissected and divided. One half was quickly emerged into an ice-cold transport buffer (BIOPS; composition (mM): CaK_2_EGTA 2.77, K_2_EGTA 7.23, Na_2_ATP 5.77, MgCl_2_·6H_2_O 6.56, Taurine 20, Na_2_Phosphocreatine 15, Imidazole 20 mm, Dithiothreitol 0.5, MES 50, pH 7.1; kept between 0 and 4 °C) and prepared for cardiac fiber isolation and permeabilization.

Mitochondrial respiration was measured in permeabilized cardiac muscle fibers. Muscle fibers were dissected free of connective tissue in ice-cold BIOPS buffer using sharp forceps. After dissection, fibers were placed in ice-cold BIOPS-buffer supplemented with 50 μg mL^−1^ Saponin for 30 min to ensure permeabilization. Fibers were subsequently washed by agitation in ice-cold MiR05-buffer (composition (mM): EGTA 0.5, MgCl_2_·6H_2_O 3.0, K-lactobionate 60, Taurine 20, KH_2_PO_4_ 10, HEPES 20, Sucrose 110, BSA 1 g L^−1^, pH 7.1) for two times 10 min. Muscle fibers were then weighed and transferred to an Oxygraph (Oxygraph-2 k; Oroboros, Innsbruck, Austria) for high-resolution respirometry. Two Substrate-Uncoupler-Inhibitor-Titration-protocols were used and all measurements were performed in duplicate. All respiratory measurements were conducted under hyperoxide conditions (200–450 nmol/mL) to ensure that oxygen would not be the limiting factor. The chosen oxygen level interval was based on recommendations from the Oxygraph manufacturer.

*Carbohydrate oxidation* State 2 respiration (GM) was assessed with malate (2 mM) and glutamate (10 mM). State 3 respiration (GM3) was achieved by addition of ADP (5 mM). Mitochondrial function was expressed as respiratory control ratio (RCR). We chose GM3/GM as RCR to examine the impact on mitochondrial respiration. Lastly, outer mitochondrial membrane integrity was controlled by cytochrome *c* (10 μM) as more than 10% increase in respiration led to exclusion.

*Fatty acid oxidation* Malate (2 mM) was added to give fundamental stimulation of complex I (state 2 respiration) followed by multiple titrations of Octanoyl-1-carnitine to achieve maximal β-oxidation and complex II respiration (MOc). State 3 respiration (MOc3) is achieved by adding ADP (5 mM) and RCR is defined as MOc3/MOc. Finally, cytochrome c (10 μM) was added to examine outer membrane integrity.

### Statistical analysis

All data are reported as mean ± SEM. Diabetic and non-diabetic groups were compared overall using two-way analysis of variance (ANOVA). Two-way ANOVA was also used to compare IR-subjected groups to SHAM, and infarct sizes across age groups. Differences in infarct sizes and RCR between groups were analyzed with one-way ANOVA with a post hoc Bonferroni test. Preischemic and ischemic hemodynamic measures were analyzed by one-way ANOVA, whereas hemodynamic measures during reperfusion were compared by two-way ANOVA. Analyses were performed using GraphPad Prism 8 (GraphPad Software, CA, USA). A *p* < 0.05 was considered statistically significant.

The number of animals in each group was *n* = 6–9 based on previous studies performed in our laboratory [26]. Power was based on reaching a significant result of infarct size with a significance level of 5% and a power of 0.80.

### Exclusion criteria

Rats were excluded according to the following exclusion criteria. Pre-protocol: illness leading to termination, divergent phenotype, unsuccessful surgical procedure and hemodynamic arrhythmias during stabilization. Per-protocol: inadvertent preconditioning, faulty protocol and high coronary flow (> 20 mL/min) compatible with unsuccessful cannulation of the heart. Post-protocol: increase in mitochondrial respiration exceeding 10% after cytochrome* c* addition.

## Results

### Baseline characteristics

Table 1 shows bodyweight, heart weight, heart weight/bodyweight ratio and fasting blood glucose levels for the rats in the infarct size study, while Online Resource 2 summarizes the same variables for the rats in the mitochondrial function study.

**Table 1:** A schematic overview of animal characteristics in the infarct size study (I) at the stages prediabetes (6 weeks), onset diabetes (12 weeks) and mature diabetes (24 weeks)

Type	Non-diabetic			Prediabetes		
Group	Con (*n* = 8)	DiMal 0.1 mM (*n* = 7)	DiMal 0.6 mM (*n* = 7)	Con (*n* = 8)	DiMal 0.1 mM (*n* = 8)	DiMal 0.6 mM (*n* = 7)
6 weeks						
Bodyweight (BW), g	158 ± 8	173 ± 5	152 ± 3	220 ± 7*	199 ± 6	213 ± 11
Heartweight (HW), g	0.60 ± 0.02	0.66 ± 0.03	0.62 ± 0.02	0.82 ± 0.04*	0.67 ± 0.02*	0.77 ± 0.03
HW/BW ratio	0.38 ± 0.005	0.38 ± 0.01	0.41 ± 0.01	0.37 ± 0.02	0.33 ± 0.008	0.36 ± 0.005
B-glucose, mmol/L	3.4 ± 0.1	4.5 ± 0.2*	3.4 ± 0.1	5.4 ± 0.2*	5.7 ± 0.2	5.1 ± 0.1

Type	Non-diabetic			Onset diabetes		
Group	Con (*n* = 7)	DiMal 0.1 mM (*n* = 8)	DiMal 0.6 mM (*n* = 7)	Con (*n* = 7)	DiMal 0.1 mM (*n* = 8)	DiMal 0.6 mM (*n* = 7)
12 weeks						
Bodyweight (BW), g	319 ± 10	304 ± 13	306 ± 17	394 ± 15*	388 ± 17	410 ± 13
Heartweight (HW), g	1.0 ± 0.03	0.99 ± 0.02	0.98 ± 0.04	1.2 ± 0.03	1.0 ± 0.07	1.2 ± 0.03
HW/BW ratio	0.31 ± 0.005	0.33 ± 0.01	0.32 ± 0.009	0.30 ± 0.007	0.28 ± 0.02	0.30 ± 0.01
B-glucose, mmol/L	4.0 ± 0.1	5.0 ± 0.2	4.0 ± 0.1	9.9 ± 2*	9.0 ± 1	11 ± 1

Type	Non-diabetic			Mature diabetes		
Group	Con (*n* = 8)	DiMal 0.1 mM (*n* = 8)	DiMal 0.6 mM (*n* = 8)	Con (*n* = 9)	DiMal 0.1 mM (*n* = 8)	DiMal 0.6 mM (*n* = 8)
24 weeks						
Bodyweight (BW), g	402 ± 8	405 ± 11	407 ± 9	403 ± 12	395 ± 19	387 ± 7
Heartweight (HW), g	1.2 ± 0.05	1.2 ± 0.03	1.2 ± 0.05	1.2 ± 0.04	1.2 ± 0.02	1.2 ± 0.03
HW/BW ratio	0.29 ± 0.01	0.30 ± 0.009	0.29 ± 0.01	0.31 ± 0.008	0.30 ± 0.02	0.30 ± 0.01
B-glucose, mmol/L	4.7 ± 0.2	5.5 ± 0.2	4.6 ± 0.1	15 ± 1*	25 ± 0.7*	13 ± 1

*p < 0.05 compared to control. Results are mean ± SEM

Body weight and heart weight did not differ between groups. Blood glucose concentrations were within normal range (< 7 mmol/L) in 6 weeks old rats and significantly elevated in diabetic rats at all ages (*p* < 0.0001).

### Infarct size

#### Diabetic vs. non-diabetic controls

**Fig. 2:**
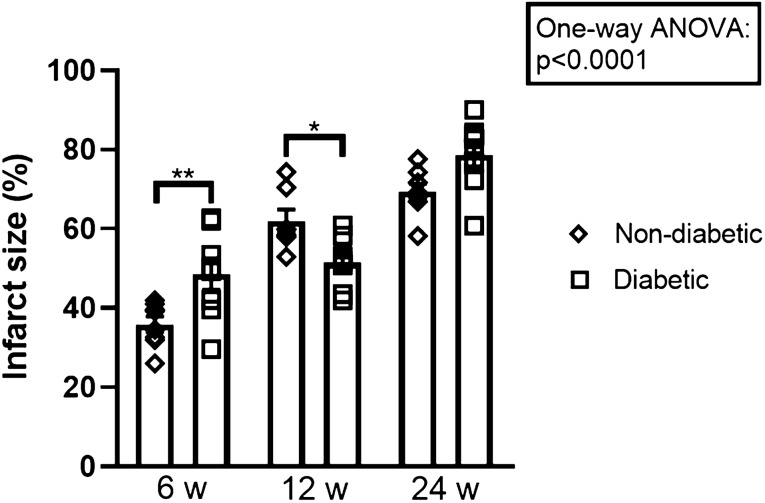
Infarct sizes for control groups. Comparisons between non-diabetic and diabetic controls at the stages prediabetes, onset diabetes and mature diabetes. *p < 0.05, **p < 0.01. Results are mean ± SEM

Infarct size increased with age in non-diabetic and diabetic rat hearts (*p* < 0.0001). In 6 weeks old rats infarct size was significantly larger in prediabetic rats than in non-diabetic rats following IR (49 ± 4% vs. 36 ± 2%, *p* = 0.007) (Fig. 2). In 12 weeks old rats, infarct size was lower at onset diabetes than in non-diabetic rats (51 ± 3% vs. 62 ± 3%, *p* < 0.05). As reported earlier, infarct size was larger in mature diabetes rats than in non-diabetic rats (79 ± 3% vs. 69 ± 2%, *p* = 0.06) in 24 weeks old rats [28], albeit not statistically significant.

#### Effect of DiMal

**Fig. 3:**
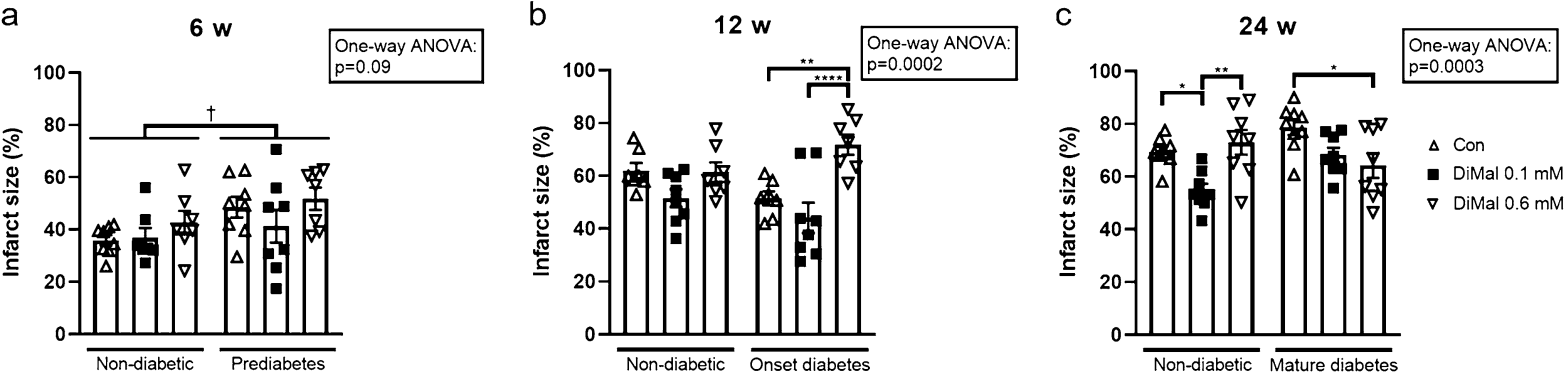
Infarct sizes in diabetic and non-diabetic rats. **a** Prediabetes and non-diabetic (6 weeks of age). **b** Onset diabetes and non-diabetic (12 weeks of age). **c** Mature diabetes and non-diabetic (24 weeks of age). For all groups infarct size (IS) is presented as a percentage of area at risk (AAR). *p < 0.05, **p < 0.01, ****p < 0.0001 (one-way ANOVA). †*p* < 0.05 (two-way ANOVA). Results are mean ± SEM

In 6 weeks old rats, DiMal did not affect infarct size in either the prediabetic or the non-diabetic group (*p* = 0.09) (Fig. 3a).

In 12 weeks old rats, neither DiMal 0.1 mM nor DiMal 0.6 mM changed infarct size in non-diabetic animals (*p* = 0.2 and *p* > 0.99). In hearts with onset diabetes DiMal 0.1 mM did not change infarct size (44 ± 6% and 51 ± 3%, *p* = 0.6), whereas DiMal 0.6 mM increased infarct size (72 ± 4% vs. 51 ± 3%, *p* = 0.003) (Fig. 3b).

In 24 weeks old rats, DiMal 0.1 mM reduced infarct size in non-diabetic hearts (55 ± 3% vs. 69 ± 2%, *p* = 0.03), while DiMal 0.6 mM did not change infarct size (73 ± 5% and 69 ± 2%, *p* > 0.99). In mature diabetic hearts DiMal 0.1 mM reduced infarct size but not significantly (68 ± 3% vs. 79 ± 3%, *p* = 0.1), whereas DiMal 0.6 mM reduced infarct size significantly (64 ± 5% vs. 79 ± 3%, *p* = 0.01) (Fig. 3c).

### Mitochondrial respiratory capacity

#### SHAM vs. IR-subjected hearts

IR reduced carbohydrate oxidation RCR compared to SHAM operated hearts regardless of diabetes status at all ages (*p* = 0.0007, *p* < 0.0001 and *p* < 0.0001, respectively) (Online Resource 3).

#### Diabetes vs. non-diabetes

**Fig. 4:**
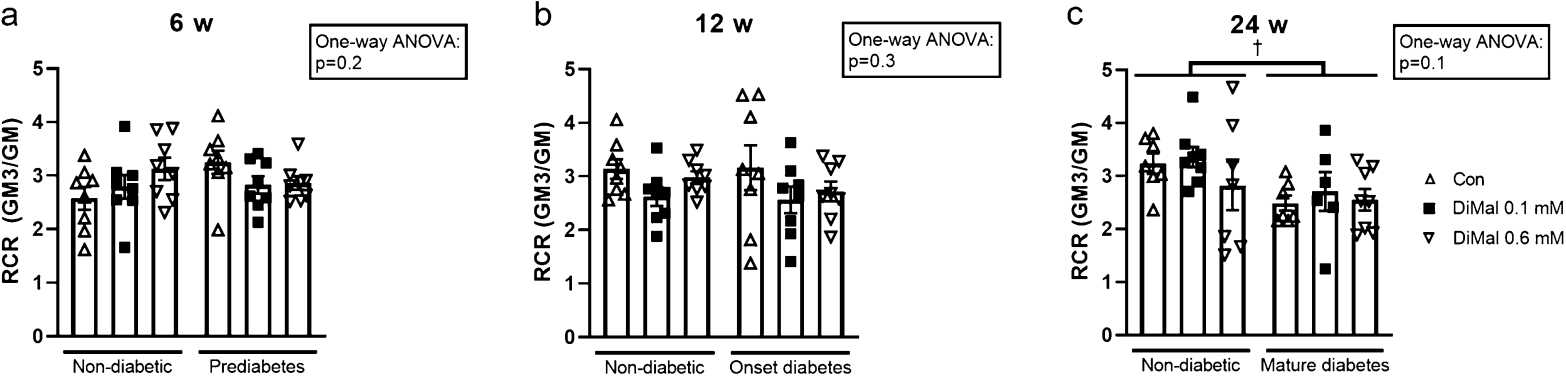
Carbohydrate respiratory control ratio (RCR) in non-diabetic and diabetic rats. **a** Prediabetes and non-diabetic (6 weeks of age). **b** Onset diabetes and non-diabetic (12 weeks of age). **c** Mature diabetes and non-diabetic (24 weeks of age). RCR is calculated as GM3/GM. †*p* < 0.05 (two-way ANOVA). Results are mean ± SEM

We found no differences in carbohydrate oxidation RCR between age-matched hearts with and without diabetes (*p* = 0.4) in the SHAM groups (Online resource 4). After IR we found no difference in RCR between prediabetic and onset diabetes and their respective control groups (*p* = 0.2 and *p* = 0.3) (Fig. 4a + b). However, at mature diabetes RCR was significantly reduced after IR compared to non-diabetic rats subjected to IR (*p* = 0.02) (Fig. 4c). Similar results were obtained with fatty acid oxidation (*p* = 0.01) (Online resource 5).

#### Effect of DiMal

In 6-, 12- and 24-weeks old rats, DiMal 0.1 mM and DiMal 0.6 mM had no effect on carbohydrate oxidation RCR in neither non-diabetic nor diabetic rats (Fig. 4). In 6- and 12-weeks old rats DiMal administration resulted in no effect on fatty acid oxidation RCR, whereas DiMal 0.1 mM resulted in significant RCR reduction in 24 weeks old rats, irrespective of phenotype (Online Resource 5). DiMal 0.1 mM reduced fatty acid oxidation RCR in non-diabetic hearts (2.2 ± 0.13 and 1.5 ± 0.084, *p* < 0.0001) and in mature diabetic hearts (1.88 ± 0.081 and 1.52 ± 0.059, *p* = 0.01).

## Discussion

The present study confirms that susceptibility to IR injury depends on stage of T2DM with increased injury in hearts with mature diabetes and decreased susceptibility at onset of diabetes [26, 27, 30]. Diabetes was not associated with compromised mitochondrial respiratory capacity during non-ischemic conditions, i.e. SHAM operation at any diabetes stage. Conversely, mature diabetes was associated with mitochondrial dysfunction following an IR challenge, demonstrating that myocardial mitochondria are more susceptible to IR due to an inherent mitochondrial disarray of mature diabetes [19, 20]. Mitochondrial dysfunction may explain altered sensitivity to cardioprotective strategies in experimental models of mature diabetes mellitus. Our findings also demonstrate that modulation of SDH activity may result in variable infarct size reduction depending not only on the presence but also on the stage of diabetes. Thus, it was unexpected that mitochondrial function with and without DiMal treatment did not differ between non-diabetic and diabetic hearts and that the effect of DiMal on mitochondrial function was not dependent on diabetes stage.

We evaluated two different doses of DiMal treatment at each stage of diabetes to clarify the effect on infarct size. Our results show that the DiMal dose required to reduce infarct size is influenced by the presence and duration of diabetes. DiMal 0.1 mM did not alter infarct size in either control or diabetic rats at 6 and 12 weeks of age, while DiMal 0.6 mM increased infarct size at 12 weeks of age in rats with onset diabetes only. Because these rats were inherently protected, high dose DiMal seems to abrograte the endogenous cardioprotection, suggesting that the intrinsic protective effect is related to SDH activity [31]. In 24 weeks old animals the reduced infarct size in non-diabetic rats with low dose DiMal (0.1 mM) confirms that SDH inhibition is cardioprotective and also that the therapeutic range is narrow as high dose DiMal (0.6 mM) abrogated the protective effect. Involvement of SDH and altered SDH activity in mature diabetic hearts is demonstrated by a differential response and a dose dependent reduction by DiMal.

During ischemia SDH activity is reversed resulting in an accumulation of succinate [16]. Upon reperfusion SDH is suggested to initiate a rapid oxidation of the accumulated succinate that overloads the capacity of the electron transport chain and gives rise to reverse electron transport through complex I and enhanced ROS production [16]. Administration of DiMal prior to and during ischemia to limit succinate accumulation [16, 17] or immediately at onset of reperfusion to inhibit rapid oxidation of succinate seems to be equally effective [18, 32]. The main focus of this study is the effect of diminishing succinate accumulation during ischemia by preischemic administration of DiMal. The ischemic period is conducted as global no-flow ischemia in the isolated perfused heart model. As DiMal is poorly metabolized by cells, administration of DiMal prior to ischemia will most probably result in DiMal being present in the cardiac tissue at initiation of reperfusion, at which point it limits the rapid oxidation of succinate [28]. However, the design of our study allows no conclusion about a potential additional beneficial effect by extending DiMal administration into onset of reperfusion.

DiMal is a synthetic agent and has the disadvantage of a narrow therapeutic range [17, 28]. Our results demonstrate that the range varies between diabetic and non-diabetic individuals. The clinical potential may therefore be limited, which is emphasized by the compound being toxic to non-ischemic tissue, such that SDH inhibition induces cell death [33]. Hence, our study serves as a proof of concept study to clarify whether adjustment of mitochondrial function by SDH modulation is feasible. Methods to limit off-target delivery and side effects might become available [17] and physiological agents might be useful alternatives to DiMal. Physiological SDH-inhibitors such as oxaloacetate and malate are intermediates of the tricarboxylic acid cycle (TCA-cycle) and natural parts of the cell metabolism. Oxaloacatete has demonstrated neuroprotective properties against IR injury in the rat brain [34]. However, it is unclear whether oxaloacetate administration is of physiological relevance in the heart [35].

Restriction of mitochondrial ROS production through TCA-cycle manipulation with DiMal has previously been raised as a potential treatment against IR injury, because SDH modulation has demonstrated infarct size reduction in experimental settings [16, 32, 36], including diabetic rat hearts [17, 28]. Diabetes modifies cellular metabolism not only due to altered fuel metabolism [25]. Increased mitochondrial uncoupling and excessive production of ROS characterize both insulin deficiency and resistance, despite their association with a normal to high oxygen consumption [21]. Although such findings have predominantly been identified in skeletal muscle, accumulating evidence indicate that T2DM is also associated with myocardial insulin resistance and decreased maximal mitochondrial respiratory capacity in experimental [37, 38] as well as in human studies [19, 21]. One potential mechanism of mitochondrial dysfunction in diabetes is imbalance between the production of ROS and capacity of antioxidants [18, 22, 39]. We found no reduction in maximal mitochondrial respiratory capacity between diabetic and non-diabetic rats as evaluated in the SHAM operated groups irrespective of the duration of diabetes. Despite varying diabetes duration in our study groups, one potential explanation may be that none of our animals had a duration of diabetes comparable to a human population with T2DM, e.g. patients with a T2DM duration of > 10.5 years have a higher probability of having an adverse cardiovascular event, indicating that irreversible damage may evolve after a longer duration of T2DM [40].

The mechanism underlying the differential response to DiMal between diabetic and non-diabetic rats remains unknown. In our study high dose DiMal had a detrimental effect on the infarct size in hearts at onset diabetes, potentially reflecting that baseline SDH activity is not increased. The activity of SDH has previously been measured in vitro at three different stages of diabetes (6, 12 and 19 weeks) and demonstrated no differences in activity compared to lean controls [41]. SDH activity has also been tested in hearts from rats with mature diabetes and age-matched controls after subjection to IR with and without DiMal with no differences between any groups [28]. Consequently, it seems unlikely that SDH activity is permanently upregulated in mature diabetes even though it would explain the increased tolerance to and enhanced effect of the SDH inhibitor DiMal at high dose. Whether SDH activity in cardiac mitochondria is temporarily modulated by the stage of diabetes in vivo is unknown. SDH activity in rat liver mitochondria is reduced one week after streptozotocin induced diabetes and normalized after one month of diabetes duration, whereas enzyme activity in rat kidney mitochondria was normal after one week of diabetes but increased after one month [42, 43]. Further studies are required to clarify whether cardiac SDH activity varies with diabetes duration.

Our study shows that SDH modulation may be challenging due to the variable response related to diabetes. Other types of comorbidity may further influence the response [44, 45]. Local delivery of DiMal to the target may limit off-target adverse effects. Previously, intracoronary administration of DiMal in pigs has exerted cardioprotective effects with undetectable concentrations in plasma and distant myocardium [36].

Limitations to the study should be acknowledged. The ZDF rat strain has a mutation in the leptin receptor and presents with a phenotype similar to that of humans with T2DM. In contrast to humans with T2DM, ZDF rats have high levels of circulating leptin, which might modulate cardioprotective pathways [44]. However, the ZDF rat is a well characterized animal model for experimental studies of T2DM [27, 46–48]. We have previously thoroughly characterized data on circulating glucose, insulin, triglyceride and total cholesterol levels. Since the patterns of blood glucose levels in the current study are similar to our previous studies, we did not repeat circulating insulin concentrations [26, 27]. The concentrations of DiMal were chosen based on results of dose–response experiments conducted in our laboratory on non-diabetic ZDF animals aged 24 weeks [28]. Diabetic animals did not receive treatment for diabetes, confining potential interactions with anti-diabetic drugs [49, 50]. The cardioprotective effects were examined using an isolated, perfused heart model. Our buffer did not contain insulin or free fatty acids, limiting resemblance of human physiology. Isolation of the heart prevented any systemic and humoral influences on the results.

In conclusion, modulation of SDH activity results in variable infarct size reduction depending not only on the presence but also on the stage of diabetes. Even though changes in cardioprotective efficacy were not associated with demonstrable alterations of mitochondrial respiratory capacity, modulation of SDH activity may be a challenging cardioprotective approach.

## Supplementary Information

Below is the link to the electronic supplementary material.

Supplementary file1 (PDF 240 kb)

Supplementary file2 (PDF 126 kb)

Supplementary file3 (PDF 197 kb)

Supplementary file4 (PDF 126 kb)

Supplementary file5 (PDF 190 kb)

## Data Availability

Authors can confirm that all relevant data are included in the article or its supplementary information files**.**
