# Supplementary material for: Myocardial salvage by succinate dehydrogenase inhibition in ischemia–reperfusion injury depends on diabetes stage in rats
Source: Mol Cell Biochem. 2021 Mar 5;476(7):2675–84. doi: 10.1007/s11010-021-04108-2 (PMC8192402; doi:10.1007/s11010-021-04108-2)
Supplement: Supplementary file 1 — Supplementary file1 (PDF 240 kb) [file 11010_2021_4108_MOESM1_ESM.pdf]

Myocardial salvage by succinate dehydrogenase inhibition in ischemia-reperfusion injury depends on diabetes stage in rats; Molecular and Cellular Biochemistry; Pernille Tilma Tonnesen, Marie Vognstoft Hjortbak, Thomas Ravn Lassen, Jacob Marthinsen Seefeldt, Hans Erik Bøtker, and Nichlas Riise Jespersen; Department of Cardiology, Aarhus University Hospital, Palle Juul-Jensens Boulevard 99, Aarhus, Denmark; pernille.tilma@clin.au.dk

Online resource 1. A schematic overview of hemodynamic parameters preischemically, during ischemia and at postischemic reperfusion.

Left ventricular developed pressure [mmHg]								
**Age/T2DM**	Intervention	n	Baseline	DiMal /no DiMal	Ischemia	Reperfusion 5 min	Reperfusion 30 min	Reperfusion 100 min
**6 weeks**								
Non-diabetic								
	Control	8	138 ± 5	135 ± 4	0.9 ± 0.1	12 ± 3	73 ± 4	59 ± 2
	DiMal 0.1 mM	7	140 ± 5	138 ± 9	0.8 ± 0.1	8.7 ± 2	54 ± 9	50 ± 3
	DiMal 0.6 mM	7	144 ± 6	139 ± 5	1.1 ± 0.06	7.6 ± 4	59 ± 7	49 ± 6
Prediabetes								
	Control †††	8	152 ± 5	148 ± 6	0.9 ± 0.2	9.2 ± 3	48 ± 6	39 ± 6
	DiMal 0.1 mM	8	148 ± 4	141 ± 7	1.7 ± 0.3*	6.4 ± 2	47 ± 5	39 ± 4
	DiMal 0.6 mM	7	165 ± 7	150 ± 4	0.7 ± 0.08	11 ± 4	42 ± 8	39 ± 4
**12 weeks**								
Non-diabetic								
	Control	7	150 ± 4	157 ± 4	1.6 ± 0.3	12 ± 4	30 ± 10	44 ± 4
	DiMal 0.1 mM ††	8	156 ± 6	146 ± 5	1.6 ± 0.2	30 ± 11	65 ± 6	52 ± 4
	DiMal 0.6 mM	7	148 ± 7	150 ± 7	1.1 ± 0.2	15 ± 3	44 ± 12	41 ± 7
Onset diabetes								
	Control	7	153 ± 23	128 ± 24	1.1 ± 0.3	12 ± 2	25 ± 9	32 ± 7
	DiMal 0.1 mM ††	8	148 ± 17	147 ± 8	0.8 ± 0.06	16 ± 5	61 ± 14	55 ± 9
	DiMal 0.6 mM	7	155 ± 13	146 ± 16	0.8 ± 0.3	12 ± 3	23 ± 5	24 ± 9
**24 weeks**								
Non-diabetic								
	Control	8	143 ± 13	136 ± 14	0.7 ± 0.1	10 ± 2	24 ± 7	32 ± 8
	DiMal 0.1 mM	8	174 ± 4	162 ± 4	0.9 ± 0.07	12 ± 4	28 ± 8	38 ± 6
	DiMal 0.6 mM	8	152 ± 8	152 ± 5	0.4 ± 0.1	14 ± 3	16 ± 6	24 ± 6
Mature diabetes								
	Control	9	160 ± 5	162 ± 5	0.7 ± 0.05	13 ± 2	21 ± 5	17 ± 7
	DiMal 0.1 mM	8	144 ± 7	128 ± 13	0.8 ± 0.09	8 ± 1	23 ± 5	29 ± 4
	DiMal 0.6 mM †††	8	170 ± 11	171 ± 9	0.8 ± 0.06	17 ± 3	42 ± 9	46 ± 5

Heart rate [bpm]								
**Age/T2DM**	Intervention	n	Baseline	DiMal /no DiMal	Ischemia	Reperfusion 5 min	Reperfusion 30 min	Reperfusion 100 min
**6 weeks**								
Non-diabetic								
	Control	8	241 ± 18	253 ± 19	174 ± 42	269 ± 26	236 ± 17	248 ± 24
	DiMal 0.1 mM	7	266 ± 22	275 ± 19	142 ± 46	271 ± 15	272 ± 13	277 ± 13
	DiMal 0.6 mM	7	239 ± 26	238 ± 28	235 ± 63	245 ± 27	296 ± 15	262 ± 9
Prediabetes								
	Control	8	247 ± 13	259 ± 14	330 ± 58	243 ± 18	278 ± 11	275 ± 12
	DiMal 0.1 mM	8	241 ± 23	252 ± 16	283 ± 19	282 ± 12	247 ± 16	258 ± 16
	DiMal 0.6 mM	7	205 ± 25	213 ± 24	222 ± 62	233 ± 27	242 ± 11	289 ± 24
**12 weeks**								
Non-diabetic								
	Control	7	201 ± 32	222 ± 16	294 ± 54	325 ± 40	238 ± 28	243 ± 14
	DiMal 0.1 mM	8	251 ± 9	255 ± 7	305 ± 13	291 ± 26	244 ± 24	279 ± 13
	DiMal 0.6 mM	7	200 ± 14	193 ± 18	211 ± 47	234 ± 19	279 ± 35	267 ± 19
Onset diabetes								
	Control	7	183 ± 38	245 ± 45	331 ± 64	239 ± 24	238 ± 43	261 ± 45
	DiMal 0.1 mM	8	157 ± 31	162 ± 13	178 ± 39	239 ± 20	190 ± 30	242 ± 32
	DiMal 0.6 mM	7	181 ± 14	185 ± 25	249 ± 60	243 ± 23	216 ± 17	271 ± 25
**24 weeks**								
Non-diabetic								
	Control	8	230 ± 15	260 ± 31	234 ± 50	263 ± 23	233 ± 17	260 ± 19
	DiMal 0.1 mM	8	212 ± 16	221 ± 16	234 ± 36	289 ± 27	233 ± 18	253 ± 18
	DiMal 0.6 mM	8	210 ± 7	193 ± 12	158 ± 55	305 ± 30	244 ± 19	290 ± 15
Mature diabetes								
	Control	9	148 ± 13 ***	175 ± 26 *	289 ± 60	179 ± 38	187 ± 29	244 ± 29
	DiMal 0.1 mM	8	150 ± 12	194 ± 14	231 ± 64	185 ± 29	218 ± 32	190 ± 34
	DiMal 0.6 mM	8	124 ± 13	124 ± 14	219 ± 41	186 ± 39	134 ± 28	235 ± 32

Rate pressure product [mmHg/min]								
**Age/T2DM**	Intervention	n	Baseline	DiMal /no DiMal	Ischemia	Reperfusion 5 min	Reperfusion 30 min	Reperfusion 100 min
**6 weeks**								
Non-diabetic								
	Control	8	33346 ± 2847	34141 ± 2690	182 ± 51	3315 ± 944	17048 ± 1261	14476 ± 1435
	DiMal 0.1 mM	7	36846 ± 2717	37175 ± 2166	125 ± 56	2349 ± 520	14844 ± 2669	13841 ± 713
	DiMal 0.6 mM	7	33877 ± 3078	32653 ± 3291	249 ± 69	1477 ± 680	17128 ± 1605	12601 ± 1303
Prediabetes								
	Control†	8	37460 ± 1703	38182 ± 2026	297 ± 67	2070 ± 620	13042 ± 1441	10863 ± 1912
	DiMal 0.1 mM	8	35751 ± 3575	35253 ± 2524	509 ± 104	1687 ± 538	11278 ± 1348	9778 ± 970
	DiMal 0.6 mM	7	32922 ± 2752	31712 ± 3265	162 ± 52	2351 ± 730	10378 ± 2196	10803 ± 608
**12 weeks**								
Non-diabetic								
	Control	7	30698 ± 4963	34591 ± 2132	497 ± 149	3393 ± 1004	7608 ± 2629	10898 ± 1236
	DiMal 0.1 mM ††	8	39120 ± 1345	37189 ± 1402	488 ± 54	8084 ± 2800	16013 ± 2605	13960 ± 1130
	DiMal 0.6 mM	7	29406 ± 1970	28626 ± 2368	272 ± 87	3342 ± 639	10926 ± 2586	10755 ± 1724
Onset diabetes								
	Control	7	23003 ± 2533	25327 ± 3625	400 ± 99	2657 ± 432	4338 ± 1162	8910 ± 2518
	DiMal 0.1 mM	8	20126 ± 2274	23954 ± 2489	147 ± 40	3403 ± 726	9420 ± 2382	12358 ± 2648
	DiMal 0.6 mM	7	27505 ± 2362	24948 ± 2019	339 ± 106	2841 ± 893	4575 ± 1020	6196 ± 2519
**24 weeks**								
Non-diabetic								
	Control	8	32566 ± 3692	35652 ± 6591	158 ± 35	2624 ± 851	5021 ± 1220	8370 ± 2050
	DiMal 0.1 mM	8	36594 ± 2312	35806 ± 2774	209 ± 43	3117 ± 896	6026 ± 1691	9385 ± 1627
	DiMal 0.6 mM	8	32223 ± 2656	29526 ± 2207	84 ± 28	4515 ± 1315	3572 ± 1238	6960 ± 1822
Mature diabetes								
	Control	9	23690 ± 2350	27844 ± 3818	205 ± 39	2175 ± 625	2933 ± 567	3989 ± 2114
	DiMal 0.1 mM	8	21913 ± 2552	23972 ± 2456	199 ± 63	1483 ± 351	4326 ± 756	4886 ± 815
	DiMal 0.6 mM †	8	20302 ± 1664	21209 ± 2769	167 ± 32	2947 ± 621	4187 ± 732	11222 ± 2125

Coronary flow [mL/min]								
**Age/T2DM**	Intervention	n	Baseline	DiMal /no DiMal	Ischemia	Reperfusion 5 min	Reperfusion 30 min	Reperfusion 100 min
**6 weeks**								
Non-diabetic								
	Control	8	13 ± 1	14 ± 1	-0.09 ± 0.02	12 ± 1	13 ± 1	10 ± 1
	DiMal 0.1 mM	7	12 ± 1	12 ± 2	0.3 ± 0.1	11 ± 1	11 ± 1	7 ± 1
	DiMal 0.6 mM	7	12 ± 1	12 ± 1	-0.03 ± 0.04	10 ± 0.5	12 ± 1	9 ± 0.9
Prediabetes								
	Control†††	8	18 ± 2	18 ± 1	0.3 ± 0.4	15 ± 1	17 ± 1	15 ± 2
	DiMal 0.1 mM †††	8	13 ± 2	14 ± 2	0.09 ± 0.1	12 ± 1	13 ± 1	10 ± 2
	DiMal 0.6 mM †	7	14 ± 1	15 ± 1	-0.08 ± 0.01	13 ± 1	14 ± 0.9	12 ± 1
**12 weeks**								
Non-diabetic								
	Control	7	20 ± 2	19 ± 2	-0.2 ± 0.2	13 ± 2	18 ± 1	15 ± 1
	DiMal 0.1 mM †	8	17 ± 1	18 ± 1	-0.04 ± 0.02	15 ± 0.7	14 ± 1	10 ± 0.3
	DiMal 0.6 mM	7	16 ± 1	18 ± 1	0.2 ± 0.2	15 ± 0.7	16 ± 0.7	13 ± 1
Onset diabetes								
	Control	7	14 ± 0.9	15 ± 1	-0.06 ± 0.03	15 ± 0.8	15 ± 1	11 ± 1
	DiMal 0.1 mM	8	14 ± 2	13 ± 2	0.3 ± 0.1	14 ± 1	14 ± 1	11 ± 1
	DiMal 0.6 mM †	7	19 ± 1	20 ± 1	-0.01 ± 0.06	17 ± 0.7	18 ± 1	14 ± 1
**24 weeks**								
Non-diabetic								
	Control	8	15 ± 1	15 ± 0.9	-0.1 ± 0.05	12 ± 2	15 ± 1	12 ± 0.8
	DiMal 0.1 mM	8	17 ± 1	19 ± 1	0.003 ± 0.2	15 ± 0.8	16 ± 0.8	11 ± 0.7
	DiMal 0.6 mM	8	15 ± 0.5	16 ± 0.6	-0.3 ± 0.07	14 ± 0.6	16 ± 0.8	12 ± 0.8
Mature diabetes								
	Control	9	13 ± 0.9	13 ± 0.7	-0.08 ± 0.05	11 ± 0.6	12 ± 0.8	10 ± 0.6
	DiMal 0.1 mM †††	8	18 ± 2	19 ± 3 *	0.06 ± 0.2	15 ± 2	16 ± 2	14 ± 2
	DiMal 0.6 mM †	8	14 ± 0.9	14 ± 0.8	-0.06 ± 0.03	13 ± 1	16 ± 2	14 ± 2

Baseline represents data measured 29 minutes into protocol, DiMal/no DiMal is collected at 39½ minutes, ischemia at 60 minutes and reperfusion is indicated as 5, 30 and 100 minutes from start of reperfusion 80 minutes into protocol. * $p<0.05$ *** $p<0.001$ as compared to control (one-way ANOVA) † $p<0.05$ †† $p<0.01$ ††† $p<0.001$ as compared to control (two-way ANOVA on reperfusion measures). Results are mean ± SEM.
