# Supplementary material for: Myocardial salvage by succinate dehydrogenase inhibition in ischemia–reperfusion injury depends on diabetes stage in rats
Source: Mol Cell Biochem. 2021 Mar 5;476(7):2675–84. doi: 10.1007/s11010-021-04108-2 (PMC8192402; doi:10.1007/s11010-021-04108-2)
Supplement: Supplementary file 2 — Supplementary file2 (PDF 126 kb) [file 11010_2021_4108_MOESM2_ESM.pdf]

Myocardial salvage by succinate dehydrogenase inhibition in ischemia-reperfusion injury depends on diabetes stage in rats; Molecular and Cellular Biochemistry; Pernille Tilma Tonnesen, Marie Vognstoft Hjortbak, Thomas Ravn Lassen, Jacob Marthinsen Seefeldt, Hans Erik Bøtker, and Nichlas Riise Jespersen; Department of Cardiology, Aarhus University Hospital, Palle Juul-Jensens Boulevard 99, Aarhus, Denmark; pernille.tilma@clin.au.dk

Online resource 2. A schematic overview of animal characteristics in the mitochondrial function study (II) at the stages prediabetes (6 weeks), onset diabetes (12 weeks) and mature diabetes (24 weeks).

**6 weeks**								
**Type**	**Non-diabetic**				**Prediabetes**			
**Group**	SHAM (n=8)	Con (n=8)	DiMal 0.1 mM (n=8)	DiMal 0.6 mM (n=8)	SHAM (n=8)	Con (n=8)	DiMal 0.1 mM (n=8)	DiMal 0.6 mM (n=8)
**Bodyweight (BW), g**	154 ±8	159 ± 11	181 ± 8	150 ± 6	182 ± 14	219 ± 9 *	222 ± 6	205 ± 12
**B-glucose, mmol/L**	3.5 ±0.1	3.7 ± 0.3	4.4 ± 0.08	3.5 ± 0.09	4.9 ± 0.1	5.4 ± 0.2 *	6.0 ± 0.2	5.2 ± 0.2
**12 weeks**								
**Type**	**Non-diabetic**				**Onset diabetes**			
**Group**	SHAM (n=8)	Con (n=8)	DiMal 0.1 mM (n=8)	DiMal 0.6 mM (n=8)	SHAM (n=8)	Con (n=8)	DiMal 0.1 mM (n=8)	DiMal 0.6 mM (n=8)
**Bodyweight (BW), g**	289 ± 6	281 ± 15	291 ± 11	308 ± 8	373 ± 6	383 ± 4 *	350 ± 12	368 ± 8
**B-glucose, mmol/L**	3.9 ± 0.08	4.2 ± 0.08	5.0 ± 0.1	4.1 ± 0.09	10 ± 1	12 ± 1 *	11 ± 1	10 ± 1
**24 weeks**								
**Type**	**Non-diabetic**				**Mature diabetes**			
**Group**	SHAM (n=8)	Con (n=8)	DiMal 0.1 mM (n=8)	DiMal 0.6 mM (n=7)	SHAM (n=8)	Con (n=8)	DiMal 0.1 mM (n=6)	DiMal 0.6 mM (n=8)
**Bodyweight (BW), g**	413 ± 9	398 ± 6	400 ± 12	423 ±12	408 ± 16	370 ± 13	375 ± 20	392 ± 15
**B-glucose, mmol/L**	4.8 ± 0.09	5.0 ± 0.1	5.1 ± 0.2	4.7 ± 0.1	12 ± 1	13 ± 1 *	25 ± 2 *	12 ± 0.9

* p<0.05 compared to control. Results are mean ±SEM.
