# Supplementary material for: Myocardial salvage by succinate dehydrogenase inhibition in ischemia–reperfusion injury depends on diabetes stage in rats
Source: Mol Cell Biochem. 2021 Mar 5;476(7):2675–84. doi: 10.1007/s11010-021-04108-2 (PMC8192402; doi:10.1007/s11010-021-04108-2)
Supplement: Supplementary file 4 — Supplementary file4 (PDF 126 kb) [file 11010_2021_4108_MOESM4_ESM.pdf]

Myocardial salvage by succinate dehydrogenase inhibition in ischemia-reperfusion injury depends on diabetes stage in rats; Molecular and Cellular Biochemistry; Pernille Tilma Tonnesen, Marie Vognstoft Hjortbak, Thomas Ravn Lassen, Jacob Marthinsen Seefeldt, Hans Erik Bøtker, and Nichlas Riise Jespersen; Department of Cardiology, Aarhus University Hospital, Palle Juul-Jensens Boulevard 99, Aarhus, Denmark; pernille.tilma@clin.au.dk

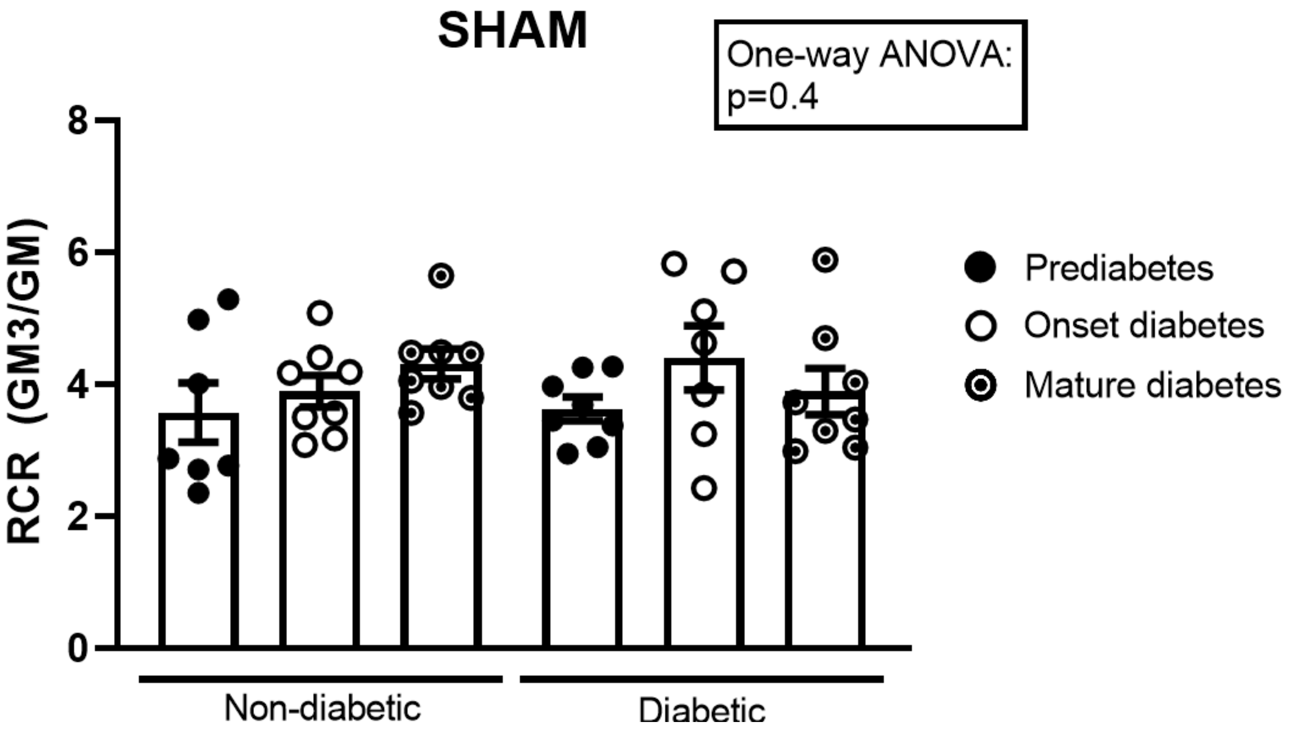

Online resource 4. Respiratory control ratio (RCR) in SHAM hearts. RCR is calculated as GM3/GM. Results are mean ± SEM.
