# Supplementary material for: Myocardial salvage by succinate dehydrogenase inhibition in ischemia–reperfusion injury depends on diabetes stage in rats
Source: Mol Cell Biochem. 2021 Mar 5;476(7):2675–84. doi: 10.1007/s11010-021-04108-2 (PMC8192402; doi:10.1007/s11010-021-04108-2)
Supplement: Supplementary file 5 — Supplementary file5 (PDF 190 kb) [file 11010_2021_4108_MOESM5_ESM.pdf]

Myocardial salvage by succinate dehydrogenase inhibition in ischemia-reperfusion injury depends on diabetes stage in rats; Molecular and Cellular Biochemistry; Pernille Tilma Tonnesen, Marie Vognstoft Hjortbak, Thomas Ravn Lassen, Jacob Marthinsen Seefeldt, Hans Erik Bøtker, and Nichlas Riise Jespersen; Department of Cardiology, Aarhus University Hospital, Palle Juul-Jensens Boulevard 99, Aarhus, Denmark; pernille.tilma@clin.au.dk

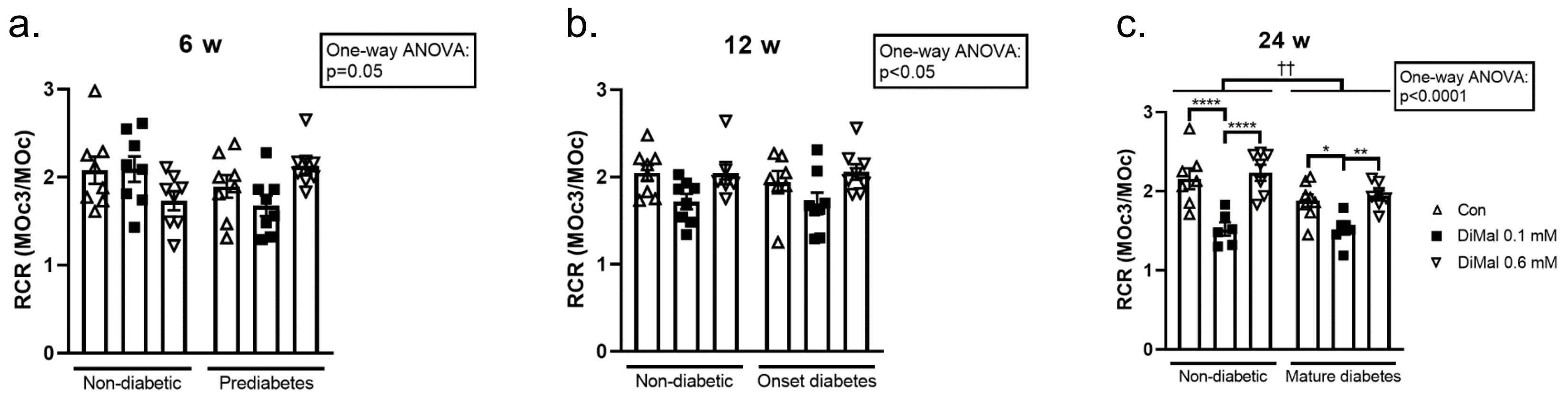

Online resource 5. Fatty acid oxidation respiratory control ratio (RCR) in non-diabetic and diabetic rats. (a) Prediabetes and non-diabetic (6 weeks of age). (b) Onset diabetes and non-diabetic (12 weeks of age). (c) Mature diabetes and non-diabetic (24 weeks of age). RCR is calculated as MOc3/MOc. †† $p<0.01$ (two-way ANOVA). Results are mean ± SEM
